# I Choose, Therefore I Like: Preference for Faces Induced by Arbitrary Choice

**DOI:** 10.1371/journal.pone.0072071

**Published:** 2013-08-16

**Authors:** Koyo Nakamura, Hideaki Kawabata

**Affiliations:** Department of Psychology, Keio University, Mita, Minato-ku, Tokyo, Japan; Inserm, France

## Abstract

Behavioral choice alters one’s preference rather than simply reflecting it. This effect to fit preferences with past choice, is known as “choice-induced preference change.” After making a choice between two equally attractive options, one tends to rate the chosen option better than they initially did and/or the unchosen option worse. The present study examined how behavioral choice changes subsequent preference, using facial images for the choice options as well as blind choice techniques. Participants rated their facial preference for each face, and chose between two equally preferred faces and subsequently rated their facial preference. Results from four experiments demonstrated that randomly chosen faces were more preferred only after participants were required to choose “a preferred face,” (in Experiment 1) but not “an unpreferred face,” (in Experiment 2) or “a rounder face” (in Experiment 3). Further, preference change was still observed after participants were informed that choices were actually random (in Experiment 4). Our findings provide new and important implications characterizing the conditions under which random choice changes preference, and show that people are tempted to make a biased evaluation even after they know that they did not make the choice for themselves.

## Introduction

Although people often believe that their preference guides choice, the idea that choice alters their preference has been an acceptable alternative [1]. This effect to fit preference with past choice is known as “choice-induced preference change,” which has been repeatedly observed. Thus, preference can be changed after making a choice between two equally attractive alternatives, as increased for chosen options and/or decreased for rejected (unchosen) options [2]–[5]. To measure this effect, previous research has used a traditional approach, referred to as a “free-choice paradigm,” where participants are asked to: (i) rate their preference for a set of items (e.g., household goods [2]) based on their desirability (pre-choice rating task); (ii) choose between two options of items that were previously rated in (i) as equal (choice task); and (iii) rate items again in the same way as in (i) (post-choice rating task). To summarize, this paradigm has shown that people tend to prefer chosen options to unchosen ones.

One possible explanation for choice-induced preference change is that having to choose between two similarly attractive options causes cognitive dissonance, which is resolved by re-evaluating the options after the choice is made. This occurrence is referred to as “cognitive dissonance theory” [6], in which cognitive dissonance is defined as an aversive state produced when people face difficulty in making decisions between similarly attractive alternatives due to conflicts stemming from the desirable aspects of the unchosen option and the undesirable aspects of the chosen option. After making a choice between two equally preferred options, one has to give up either of the two options. To cope with this situation, one tends to deliberately increase preference for the chosen option and decrease their preference for the unchosen option. For example, the idea that action alters preference is illustrated in Aesop’s Fable “The Fox and the Grapes.” In this story, a fox tries to get delicious looking grapes but finally realizes they are inaccessible, and decides to give up, convincing himself that the grapes were probably sour and questioning why he would want them. Here, the fox exhibits two types of cognition: first is the fact that he wants the grapes, and second is the fact that he cannot obtain them. According to cognitive dissonance theory, the fox embraces the dissonance between the two types of cognition by giving up on the grapes, such that he copes with this situation by changing his preference for the grapes. Thus, the dissonance can be resolved by rationalization based on logical consistency between two cognitions such as one’s own action and preference to maintain self-consistency in attitude [6], [7].

An alternative explanation of choice-induced preference change is self-perception theory. Within the theory, it is assumed that individuals come to know their own preferences by inferring them from observations of their own behavioral choices to the extent that internal cues are ambiguous [8]. According to self-perception theory, individuals learn their own preferences after making a choice between two equally attractive options, and then update their preferences in accordance with the choices. Critical difference between cognitive dissonance theory and self-perception theory is the matter of aversive psychological state. Self-perception theory postulates no aversive motivational pressure [8], while a motivation to reduce cognitive dissonance is central part of cognitive dissonance theory [6]. Despite differences in the underlying processes hypothesized in each theory, both these theories predict post-choice change in preference.

In the last few years, notable characteristics of choice-induced preference change have been empirically demonstrated. One remarkable aspect of the preference change is its stability. For example, Sharot, Fleming Yu, Koster and Dolan [9] observed that choice-induced preference changes for vacation destinations sustained 2.5 to 3 years after participants made choices. Similarly, Coppin, Delplanque, Porcherot Cayeux and Sander [10] also reported that preference changes persisted for a week after making choices even when participants did not explicitly remember which options they chose. These findings suggest that this phenomenon is stable and persistent for a long period of time once choices induced revaluation. In addition to the long-term stability, it is argued that the phenomenon can occur outside the context of normal adult human decision-making. In fact, choice-induced preference change was observed in amnesic patients who did not explicitly remember which options they chose [11], and in children and capuchin monkeys which did not fully develop highly cognitive functions [3], [12]. Taken together, these studies imply that choice-induced preference change relies upon the automatic and robust mechanisms without requiring explicit memory or highly developed cognitive functions.

Other recent studies have developed a new method to measure preference change, and indicate that choice does not necessarily need to be guided by one’s own preference [12], [13]. The mere act of choosing may be sufficient to induce preference change rather than choosing the truly preferred options [14], [15]. Indeed, Sharot, Velasquez, and Dolan [13] have demonstrated that choice-induced preference change was observed even when a choice was made randomly (the “blind choice task”). In their study, participants were simply forced to choose between dummy options regardless of their preference, while they believed that they made choices on the basis of subliminal decision-making. This procedure made participants believe that they chose the option they truly liked before actually seeing the options. The experiments also included the pre-choice rating task, the choice task, and the post-choice rating task, using a traditional free-choice paradigm [2]. To induce arbitrary selection, this choice task presents options briefly without awareness. In fact, even arbitrary choice increased preference ratings for the chosen options, but only when participants believed that the choices were based on their intention, and not when they believed the choices were made by a computer [13]. This finding suggested that even the act of choosing might enhance commitment to the chosen options, resulting in a fondness for the chosen option.

In the present study, we investigated the conditions under which arbitrary choices alter subsequent preferences, and whether preference change can be revoked once choice decisions are made. Using a blind choice task [13] in which participants made a choice without seeing the alternatives, we examined (i) whether even arbitrary choice induces preference change for face images (Experiments 1); (ii) whether method of choice (i.e., choosing a preferred or an unpreferred option between two alternatives) modulates the impact of arbitrary choice on subsequent preference (Experiments 2); (iii) whether the mere act of choice that is not directly related to preference decision-making triggers preference change by the comparison between judgments of facial preference and non-evaluative features (i.e., judging facial roundness, Experiment 3); (iv) whether the impact of choice on preference can persist even when participants are informed that their choices did not reflect their own preferences (Experiment 4).

## Experiment 1

In Experiment 1, we investigated whether choice induces preference change even when options included facial images that had not previously been examined. Previous studies have examined this post-choice change in preference by using a variety of options (i.e., stimulus types) that could be expected or experienced, such as kinds of illnesses [16], vacation destinations [4], [14], presidential candidates [17], foods [5], odors [10], [18], and household objects [2]. We tested facial images and whether preference for faces could be changed through blind choice. Faces contain important information, including many signals that evoke emotion, attractiveness, trustworthiness, and diverse social decisions such as the selection of friends and mates [19], [20].

### Method

#### Ethics statement

All experiments were approved by the local ethical committee of the Keio University, Japan. Before starting each experiment, participants individually provided informed consent and signed a written consent form. A proper debriefing was conducted for each participant at the end of our study, explaining the purpose of using deceptive instructions during a blind choice task as described hereafter.

#### Participants

Twenty adults (10 females; mean age, 21.1±0.89 years) participated in the present experiment. All participants had normal to corrected-normal vision and were not aware of the purpose of the experiment. They were individually tested and paid 1,000 Japanese yen for their participation.

#### Apparatus and stimuli

The facial stimuli were presented on a 21-inch monitor (Trinitron CPD-G420, SONY) controlled by the MATLAB program (The Math Works, Natick, MA) using a MacBook Pro (MacBook Pro, Apple). Participants sat at a viewing distance of 57 cm away from the monitor. Stimuli consisted of 240 faces generated by computer software (www.facegen.com/) in four subcategories: two races (Asian, European)×two genders (male, female). All faces were emotionally neutral, and had no hair. In our preliminary experiment, a separate group of seven participants (4 females; mean age, 20.1±1.42) rated facial attractiveness on a scale from 1 to 8 (1: not attractive at all, and 8: highly attractive). The mean scores for the attractiveness ratings of the faces were 4.15±0.40 for the male faces and 4.20±0.58 for the female faces.

#### Procedure

The present experiment was divided into three parts: a pre-choice rating task, a blind choice task, and a post-choice rating task.

The pre-choice rating task consisted of 240 trials (120 trials for female faces). In the task, participants were required to evaluate their subjective preference for faces presented on a computer screen one at a time. Face images were 7°×11° in visual angle in the center of the screen against a black background. In each trial, participants pressed the space key to initiate presentation of a face image and rated their preference for that face on a scale from 1 to 8 using numbers on their keyboard (1: not preferred, 8: highly preferred). Participants were able to view the face until a response was made in each trial (however, they were encouraged to intuitively judge the faces). Two sessions were separated according to the gender of the faces, in which the faces were presented in a random order. The order of sessions (i.e., gender of the faces) was counter-balanced across the participants.

**Figure 1:**
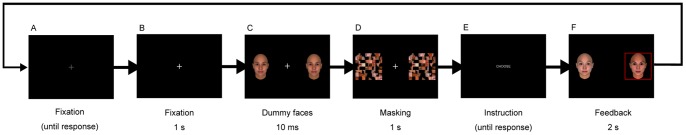
Sematic diagram of a typical trial during the blind choice task. Participants were required to press a key to initiate a trial (A), followed by a presentation of fixation for 1 s (B). Next, two identical dummy faces were presented for 10 ms (C) and followed by masking stimuli presented for 1 s (D). After the dummy faces were presented, participants were asked to respond in accordance with an instruction (e.g., “CHOOSE” represents to choose a preferred face). Once completed the response, target faces were presented (F). Note that participants were instructed that target faces were presented two times within a trial, one in C and another in F.

In the blind choice task (Figure 1), participants were instructed about each trial and told that two faces would appear on the screen side by side for an extremely short time (approximately 10 ms), that the faces would be followed by masked images, and their task was to choose the more preferred face of the two based on their intuition. As a cover story, participants were told that the task was designed to investigate “subliminal decision-making”, similar to the methodology used in Sharot et al. [13], in which participants could make a choice based on implicit preference even if they could not perceive the pair of faces due to the extreme brevity in presentation time. To ensure that participants believed the cover story, the experimenter briefly introduced a summary of research on subliminal decision-making [21], [22]. Participants were also told that after completing an intuitive choice, the two faces would reappear on the screen and they could confirm their selection of the chosen face and a red frame would appear around the chosen face, indicating that they chose it at will. In reality, however, these instructions were different from the actual stimulus presentation as described below. In fact, we presented a pair of identical faces never used in the choice task (referred to as dummy faces) for 10 ms, followed by the masking stimuli that comprised jumbled facial parts created from several images. Therefore, participants’ choices could not be guided by pre-existing preference. Next, the word “CHOOSE” appeared on the screen, instructing participants to make their decision. The choice was completed by pressing the key corresponding to each face (the one on the right or the one on the left). After making a choice, the pair of faces that had been assigned (known as target faces) were presented on the screen for 2 s, and the blindly chosen face was marked by a colored frame to emphasize the choice the participants made for themselves. During the blind choice task, there were two types of trials: critical trials and non-critical trials. In the critical trials (which occurred approximately 70% of the time), two alternatives for which the participant had given the same rating score in the pre-choice rating task were presented as the target faces. The remaining trials (non-critical trials) included two options that the participants had rated differently during the pre-choice task. To enhance the power for detecting the impact of choice during the critical trials, we assigned the more preferred face to the side that the participant chose and the less preferred face on the opposite side during the non-critical trials. So as not to choose based on rule-based strategies, participants were required not to alternate between the left and the right and also to choose one side consistently. We adopted this type of blind choice paradigm to circumvent the issue raised by Chen [23] and Chen and Risen [24], in which they noted that post-choice changes in preference may not necessarily reflect an impact of choice on preference, rather it may be due to the artifact that the preference change was merely a reflection of a participant’s preexisting preferences. In this paradigm, participants had to make choices on facial preference before they were presented. Therefore, choice cannot be guided by pre-existing preferences. The choices made during this task were used to classify the trials for the pre- and post-choice rating task into trials for subsequently chosen and unchosen stimuli. As with the pre-choice rating task, two sessions were separated according to the gender of the faces. Participants were not informed of the existence of the post-choice rating task until the trials were completed.

After completing the choice task, the participants were again required to evaluate subjective preference for facial stimuli during the post-choice rating task. In the task, all 240 faces were presented one by one in a randomized order. This was completely identical to the pre-choice rating task.

#### Data analyses

We analyzed the data in the same manner as in Sharot et al. [13]. Data from the non-critical trials were eliminated because those were not our primary interests. We calculated post-choice change in preference by subtracting the mean-corrected pre-choice rating score from the mean-corrected post-choice rating score for each participant and each facial stimulus. Next, the mean preference change score was calculated for each participant, according to the results from the chosen or unchosen on the blind choice task. Here, the mean-corrected score refers to the distance of a particular stimulus’s rating from the average rating for that participant and session (x_i_ − µ) − the value of the stimulus relative to all other stimuli in that session. We performed a one-sample *t* test to examine whether these scores were significantly different from zero (µ = 0). A paired *t* test was conducted to examine the differences in preference change between the chosen faces and unchosen faces.

### Results and Discussion

**Figure 2:**
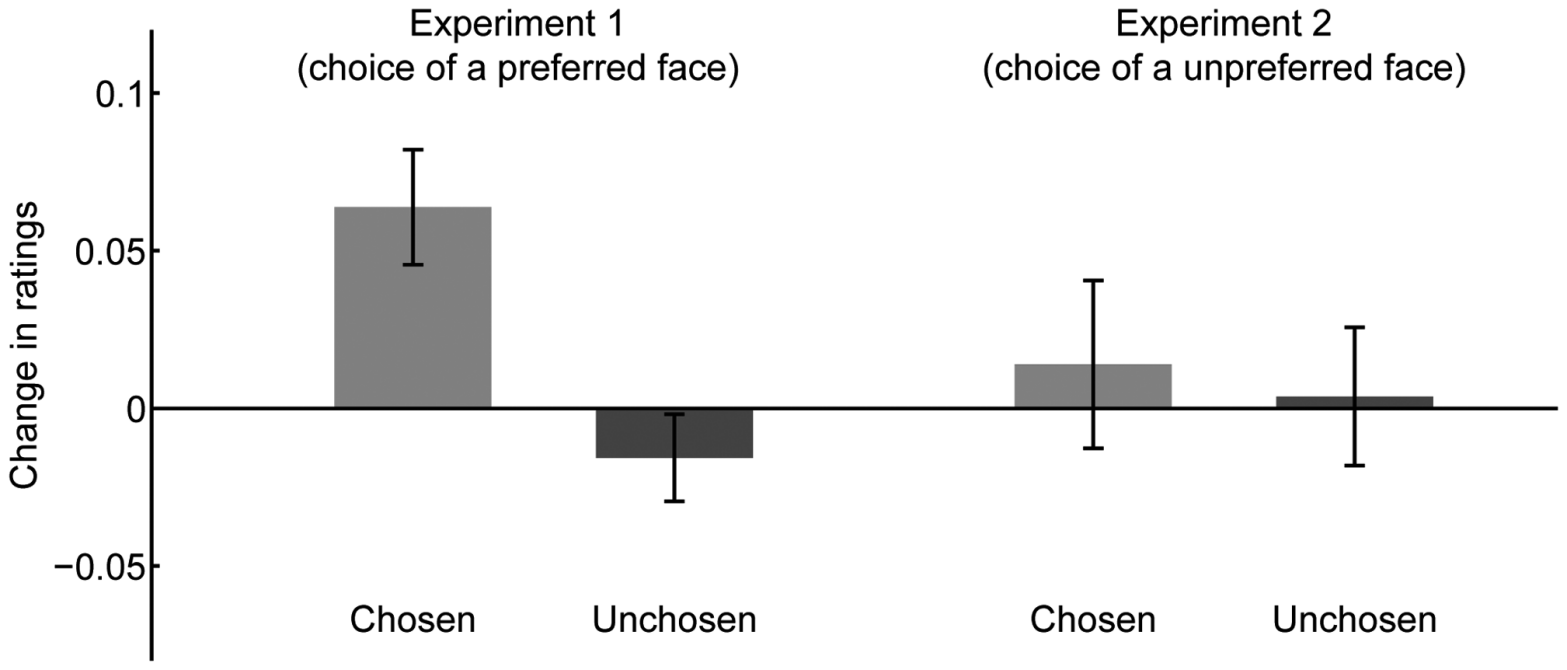
Post-choice change in preference in Experiments 1 and 2. Change in preference ratings for chosen and unchosen facial stimuli after a blind choice between two equally preferred faces in Experiment 1 (choice of a preferred face between two alternatives) and Experiment 2 (choice of an unpreferred face between two alternatives). Bars indicate differences in mean-corrected ratings between the pre- and post-choice rating tasks. Error bars represent standard errors (SE) of the mean.

The one-sample *t* test revealed preference changes for the chosen faces (Figure 2, left panel). Ratings for the chosen faces increased after the blind choice task (*t*(19) = 3.50, *p*<.005), while ratings for the unchosen faces were not modulated (*t*(19) = −1.13, *p*>.1). The significant increase in ratings for the chosen face was larger than the non-significant decrease for the unchosen faces (*t*(19) = 3.33, *p*<.005).

This experiment demonstrated that even when choices were made randomly, (that is, choices were not guided by participants’ own preference), choice shaped facial preference. Indeed, facial preference ratings for chosen options increased after making choices while ratings for unchosen options remained the same. This result was consistent with that reported by Sharot et al. [13], further showing that choice-induced preference change could apply to face options. Thus, our data suggest that the blind choice task conducted by Sharot et al. [13] may be applicable to facial stimuli.

## Experiment 2

In most previous studies, the choice task in typical free-choice paradigm asks participants to choose a more attractive or preferred option between two alternatives [4], [5], [13]. If the preference change is mediated by logical consistency between one’s own choice and preference, choosing unpreferred options can also induce preference change because choice of a preferred and an unpreferred option is semantically opposite and both reveal relative preference for each option within a binary choice situation. However, some psychological evidence has indicated that assessing an object as preferable and unpreferable is involved in different eye gaze patterns [25] and brain regions [26], leading to qualitatively different psychological processes. In Experiment 2, we examined whether choosing an unpreferred option between two alternatives induced preference change.

### Method

#### Participants

Twenty different adults (10 females; mean age, 22.8±7.46 years) participated in Experiment 2 (thus, no individuals overlapped between the two experiments). All participants had normal to corrected-normal vision. They were individually tested after taking informed consent and paid 1,000 Japanese yen for their participation.

#### Stimuli

The facial stimuli and the experimental settings were identical to those in Experiment 1.

#### Procedure

The pre-choice and post-choice rating tasks were identical to those in Experiment 1. Only the blind choice task was different from that of Experiment 1, as participants were asked to choose unpreferred faces during the blind choice task.

### Results and Discussion

A one-sample *t* test revealed no preference changes when choosing unpreferred faces (Figure 2, right panel). Rating scores after the choice task did not change for either of the chosen faces (*t*(19) = 0.51, *p*>.1), or unchosen faces (*t*(19) = 0.16, *p*>.1). Preference changes in the chosen and unchosen faces did not significantly differ (*t*(19) = 0.25, *p*>.1). These results suggest that preference-related choice does not always induce preference change. That is, post-choice change in preference was not seen in unpreferred judgment. These results cannot be fully accounted for by the cognitive dissonance theory. Cognitive dissonance theory predicts that choosing unpreferred faces induces preference change, because there are dissonant cognitions between choice of unpreferred faces and preference for faces. The results in Experiment 2 indicated that the choice between a preferred option and unpreferred option has a different implication in the psychological process in a binary choice situation despite semantic correspondence. Indeed, previous research has indicated that assessing an object as preferable and unpreferable relies on different psychological mechanisms [25]–[28]. This can be true for the impact of choice on preference formation.

However, we could not rule out the possibility that experimental artifacts ostensibly canceled out the effect of preference change in Experiment 2. Participants were asked to choose ‘preferred’ faces in Experiment 1, whereas they were asked to choose ‘unpreferred’ ones in Experiment 2. According to cognitive dissonance theory, declaring options as ‘unpreferred’ might decrease ratings for them during a post-choice task. However, our data did not show this pattern within a preference change effect. As pointed out in some previous studies, it could be that merely selecting or attending to an affectively neutral stimulus influences its subsequent affective evaluation even when participants make a decision unrelated to preference [29]–[31]. If true, some types of choice that do not seem to be directly related to preference judgment may induce preference change as well. If participants evaluate chosen options highly by merely selecting or attending to them, then viewing chosen options as unpreferred might increase their preference for them through the mere act of choice. Therefore, the impacts on decreasing preference for ‘unpreferred’ options and increasing preference for ‘chosen’ options might be cancelled out.

## Experiment 3

To test whether post-choice change in preference is triggered by the mere act of choosing that is not directly related to preferential decision-making, we conducted Experiment 3, in which participants were asked to choose a preferred face during some trials (preference judgment) and a rounder face during other trials (roundness judgment). The roundness judgment is often used as a comparative condition to preference judgment for facial attractiveness [25], [32]. We compared preference changes in choices for preferred faces with those from the choices not directly related to facial preference judgments.

### Method

#### Participants

Twenty-one different adults (10 females; mean age, 19.8±1.00 years) participated in Experiment 3 (that is, no individuals overlapped among Experiments 1, 2, and 3). Again, all participants had normal to corrected-normal vision. They were individually tested after taking informed consent and paid 1,000 Japanese yen for their participation.

#### Stimuli

The facial stimuli and the experimental settings were identical to those in Experiment 1 and 2. As a preliminary experiment, a separate group of five participants (3 females; mean age, 21.4±1.14) rated the facial roundness of the facial stimuli used in Experiment 1 on a scale from 1 to 8 (1: not round at all, and 8: extremely round). The mean scores for the roundness ratings of the faces were 4.33±1.57 for male faces and 4.83±1.39 for female faces, respectively. Facial attractiveness was based on the preliminary findings from Experiment 1.

#### Procedure

This third experiment contained the pre-choice rating, the blind choice, and the post-choice rating tasks. The pre-choice and post-choice rating tasks were identical to those in Experiments 1 and 2. Only the blind choice task differed from Experiments 1 and 2; in the blind choice task, there were two types of trials: preference judgment and roundness judgment trials. All pairs of faces were determined by a MATLAB program and were divided into these two trials in which an approximately equal number of trials for each set were included. In the preference judgment trials, participants were instructed to choose a more preferred face between two alternatives as in Experiment 1. During the trials, after briefly presenting two dummy faces, the instruction on the center of screen read “Preference.” In the roundness judgment trials, after briefly presenting two dummy faces, participants were asked to choose the rounder of the two faces when the instruction on the screen read “Roundness”. Before starting the experiment, participants were fully instructed to judge the physical roundness of faces on each pair (i.e., which face seemed to be rounder). These trials were intermixed throughout a session and the number of each trial was approximately identical. Crucially, choices during the blind choice task were not based on pre-existing preferences for both preference and roundness judgment. As with Experiment 1, approximately 70% of both the preference judgment and roundness judgment trials consisted of pairs of faces that participants had rated the same in the pre-choice rating task (critical trials). The rest of the trials during the preference judgment trials included pairs of faces differently rated by the participants. We assigned the more preferred face onto side that a participant previously chose and the less preferred face onto opposite side of the screen (during non-critical trials). Similarly, of the remaining trials during the roundness judgment consisted of pairs of faces that a separate group of participants had rated differently for facial roundness. For those trials, we assigned the rounder face onto the side that the participant previously chose and the less round face onto the opposite side (non-critical trials).

### Results and Discussion

**Figure 3:**
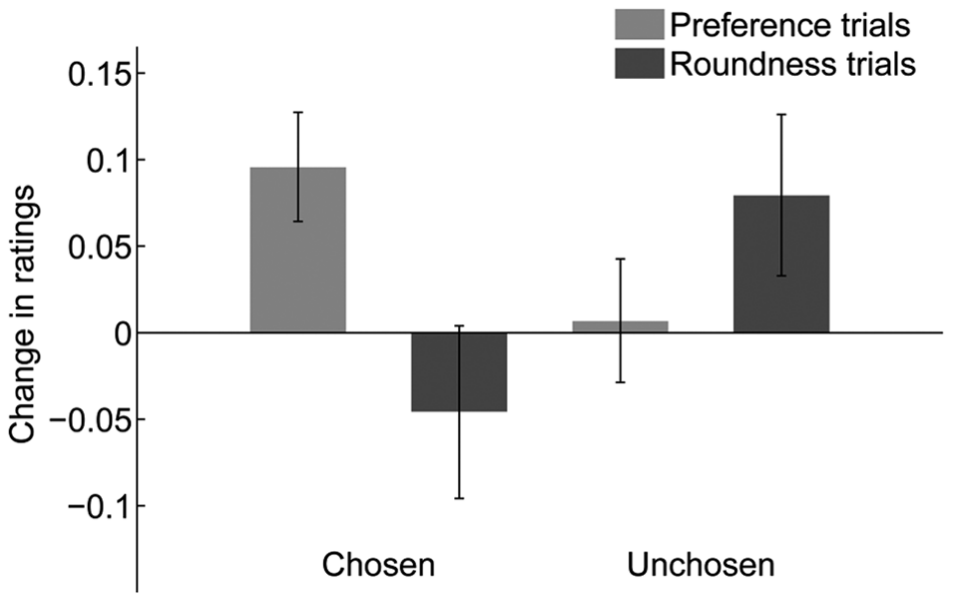
Post-choice change in preference in Experiment 3. Change in preference ratings for chosen and unchosen facial stimuli after a blind choice between two equally preferred faces in the ‘preference judgment’ trials (choice of a preferred face between two alternatives) and the ‘roundness judgment’ task (choice of a rounder face between two alternatives) in Experiment 3. Bars indicate differences in mean-corrected ratings between the pre- and post-choice rating tasks. Error bars represent standard errors (SE) of the mean.

To examine the difference in preference change as a dependent variable between preference trials and the roundness trials, we conducted a 2×2 repeated-measures ANOVA with choice (chosen, unchosen) and judgment type in a trial (preference, roundness), which were both within-subject factors. Results (Figure 3) demonstrated a significant interaction between choice and judgment type (*F*(1,20) = 10.5, *p*<.005). The simple-effect analysis revealed that change in preference for the chosen face was significantly higher than the unchosen face in preference judgment (*F*(1,40) = 4.15, *p*<.05), and that the unchosen face was significantly higher than the chosen face during the roundness judgment (*F*(1,40) = 8.26, *p*<.01). Neither the main effect for choice nor that in judgment type was statistically significant (*F*(1,20) = 0.29, *p*>.1, for choice; *F*(1,20) = 0.41, *p*>.1, for judgment type). In addition, one-sample *t* tests revealed that ratings for the chosen faces for the preference judgment trials significantly increased after making choices (*t*(20) = 3.04, *p*<.01), while those for the unchosen faces was not modulated by choice (*t*(20) = 0.19, *p*>.1). In contrast, neither post-choice change in preference for the chosen nor the unchosen during the roundness task was statistically significant (chosen, *t*(20) = −0.92 *p*>.1; unchosen, *t*(20) = 1.71 *p*>.1). That is, it indicated that ratings were changed after the blind choice only when participants responded to choose preferred faces between two alternatives, whereas post-change in preference was significant between chosen and unchosen faces for the roundness task.

These results could ensure that the mere act of choosing that is not directly related to preference decision-making was not sufficient to induce preference change. As far as our data is concerned, we assumed that enhanced commitment by a choice decision for preferred faces could produce preference change, but not the mere act of choice relatively independent of preference (i.e., roundness in this experiment). Therefore, what to choose is important for choice-induced preference change.

## Experiment 4

Experiment 1 revealed that chosen faces came to be preferred even when choices were not guided by pre-existing preferences. Here, we examined the effects when participants were informed that their choices had not reflected their own preferences, but only after the choices were made. Recent studies suggest that the update of preference occurs immediately after making a choice and its updated preference is stable [9], [11]. Given the stability of updated preference, the impact of arbitrary choices may persist even after participants were informed their choices were random. In Experiment 4, we gave participants a post hoc explanation that choices were actually not based on their preferences immediately after the blind choice task, making participants explicitly understand that preferences do not have to fit with their choices. Therefore, choice-induced preference change should not occur in such situations, if post-choice change in preference is derived from consciously fitting their preferences with their choice in the post-choice rating task.

### Method

#### Participants

Twenty-two different adults (12 females; mean age, 21.5±1.24 years) who did not participate in Experiments 1, 2, or 3 participated in Experiment 4. Again, all participants had normal to corrected-normal vision. They were individually tested after taking informed consent and were paid 1,000 Japanese yen for their participation.

#### Stimuli

The facial stimuli and the experimental settings were identical to those in Experiment 1, 2 and 3.

#### Procedure

Most parts of the experimental tasks were identical to those in Experiment 1 except for the fact that participants received debriefing for the blind choice task immediately after the choice task. Before the blind choice task, participants were instructed to choose a preferred face, although they cannot consciously perceive their options, as done in Experiment 1. After completing the choice task, the experimenter gave the participants a debriefing form describing that the procedure of the blind choice task made it impossible to choose truly preferred faces based on their own preferences and asked them to carefully read and understand the contents. The debriefing of subjects was done using both written and oral explanations. During the debriefing, the illustrative figure complemented that the two briefly presented faces (dummy faces) were indeed identical faces and crucially the two faces were always unrelated to the target faces that were presented after choice, whereby participants could not make preference decisions based on their own preferences. All of the participants could fully understand this debriefing instruction, and signed the written form and participated in the post-choice rating task.

### Results and Discussion

**Figure 4:**
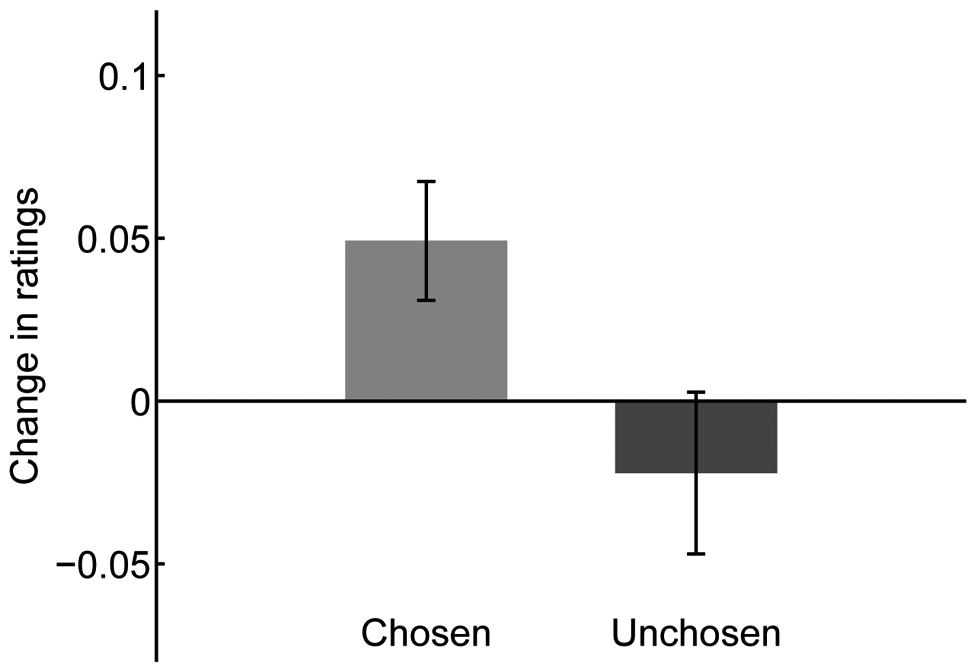
Post-choice change in preference in Experiment 4. Change in preference ratings for chosen and unchosen facial stimuli after blind choice between two equally preferred faces in the Experiment 4 (choice of a preferred face between two alternatives). Note that participants rated their preference for faces after they explicitly knew that their choices had been actually random. Bars indicate differences in mean-corrected ratings between the pre- and post-choice rating tasks. Error bars represent standard errors (SE) of the mean.

One-sample *t* tests revealed preference changes even after participants received the debriefing feedback (Figure 4). Ratings for the chosen faces increased after making choices (*t*(21) = 2.69, *p*<.05), while ratings for the unchosen faces were not modulated by choice (*t*(21) = −0.89, *p*>.1). The increase in ratings for the chosen face tended to be larger than the non-significant decrease for the unchosen faces (*t*(21) = 1.94, *p*<.1).

These results revealed that choice-induced preference change persisted even after participants explicitly understood that their choices were not based on their own preferences. In fact, randomly chosen faces were more preferred even after participants explicitly understood that preferences do not have to fit with their choices. This implies a tendency for preference change is close to that of Experiment 1, though we could not determine whether the debriefing before post-choice rating diminished the preference change effect. Future study should address whether the debriefing eliminate the effect by comparing debriefed and non-debriefed conditions within an experiment. Nevertheless, our results indicate that preference can be changed irrespective of whether participants believe that the choices for face to be true or false. One interpretation of these results are that choice-induced preference change may be irrevocable even if participants were informed that choice is actually random later, once choices trigger revaluation at the moment of choice [33].

## General Discussion

The present study revealed several important features of choice-induced preference change. Experiment 1 showed that facial preferences were altered in accordance with the mere act of randomly induced choice, suggesting that facial preference was also susceptible to the outcome of past choices. Experiments 2 and 3 demonstrated that post-choice change in preference was not observed during the unpreferred facial judgment or during non-evaluative features judgment. In fact, choice for unpreferred or rounder faces did not change preference ratings, whereas choice for preferred faces increased the preference for chosen options. Experiment 4 suggested that choice-induced preference change remained even after participants explicitly understood that their choices were actually random, regardless of whether it was based on a pre-existing preference. This can imply that revaluation is rapidly engaged and the updated preferences are maintained even after participants were informed that choices were actually random.

### Impact of Choice on Facial Preference

Choice-induced preference change has been examined using a variety of option types, such as household objects [2], vacation destinations [4], and foods [5]. However, few studies have directly investigated the impact of choice using facial stimuli, although faces are distinguishable social signals [28], [34]. In the pre- and post-choice rating tasks, participants are typically asked to rate their preference for objects, in accordance with their experienced or expected values [4], [10]. In the present study’s experiments, participants were asked to rate facial preference, which reflected experienced value, using a blind choice task. Our findings extended the results from Sharot et al. [13] measuring expected value, in which participants were asked to imagine how pleasant it would be if they could spend their vacation in a given destination. In fact, facial preference may be one strong determinant for interpersonal communication that ensures reliable partnerships and friendships among people [20], [34]. Although physical features such as symmetry and averageness [19], [35], [36] automatically influence affective response in beholders, facial preference is also susceptible to their past choice. As Sharot et al. [13] noted, post-choice change in preference would be adaptive for facilitating people’s commitment to their chosen option, allowing them to make consistent and rapid decision-making. Considering the pervasive impact of facial preference on diverse decision-making such as in the choice of a partner or friends, evaluating one’s own choice in the real world may enhance positive feelings for them and strengthen partnerships or friendships, thus contributing to healthy communication and collaboration within groups of people.

### Causes of Choice-induced Preference Change

Since Brehm’s [2] initial study, a dozen studies have accepted cognitive dissonance theory as a plausible interpretation of choice-induced preference change [3], [5], [33]. Our results also can be interpreted by cognitive dissonance theory. This theory would explain our results as follows: Observing individual’s own blind choice generates contradictory cognitions (e.g., “There are the undesirable aspects of the face” and “I chose it”) and individuals are motivated to cope with the aversive state aroused by holding these dissonant cognitions. To reduce the cognitive dissonance, participants increased their preferences for blindly chosen face (Experiment 1). When participants believed that they chose a rounder face (Experiment 3), dissonance does not arise, because their blind choice of the rounder face and their preference for the face is not inconsistent (e.g., “There are the undesirable aspects of the face” and “I blindly judge the face to be rounder”). So far, previous studies using the traditional free-choice paradigm have posited that after making a difficult choice between two equally preferred options, cognitive dissonance is experienced [2], [4]. In the blind choice task, however, no volitional choices were actually made, where participants cannot help rationalizing their alleged choice. Therefore, post-choice change in preference observed in our study might be due to an artificial dissonance. This can indicate that even mere act of choice triggers cognitive dissonance [13], [14].

Alternatively, our results can be explained by self-perception theory that does not posit the reduction of cognitive dissonance [8], in which it is assumed that individuals estimate their preferences by inferring them from observations of their own behavioral choices. In our experiments, participants believed they observed their choices guided by their implicit preferences. After making an arbitrary choice, participants observed their choice between two equally preferred facial stimuli and updated their preferences to fit with their choices. When participants believed that they chose a rounder face (Experiment 3), their preference was not updated, as the choice did not reveal their internal preference for faces. Thus, self-perception theory predicts that wrongly perceiving the blind choices as informative about their underlying preferences alters subsequent preferences for faces. This theory would provide a more parsimonious explanation for our results because it does not assume any other psychological processes than observing choice and inferring preference.

However, both theories cannot fully predict our novel observations from Experiments 1 and 2 showing that randomly chosen faces were more preferred only after participants were required to choose “a preferred face,” but not “an unpreferred face”, suggesting that preference-related choice does not always induce preference change. Even though choices for preferred and unpreferred options were semantically opposite and both revealed relative preference for each option in a binary choice situation, each of the choices influenced subsequent preference differently. This may be partially due to the fact that face is generally a positive stimulus and participants potentially evaluate how attractive someone is, but not how unattractive she or he is [28], [37]. Thus, asking people to choose unpreferred faces could be unnatural and the choice may not be registered in the same way as natural choices (i.e., choosing preferred faces) are. Moreover, some suggest that liking and disliking judgment are differently represented in memory, leading to qualitatively different effects on behavior [38], [39]. The idea that these judgments are functionally distinct constructs has been supported by biological evidences demonstrating that evaluating facial attractiveness and unattractiveness triggers different eye gaze patterns [25] and brain activities [26]–[28]. Thus, the functional separability in choosing preferred and unpreferred options might constrain impact of choice on subsequent preference formation.

In light of the functional separability, choices of unpreferred options may induce revaluation under some conditions. Indeed, Sharot et al. [16] demonstrated that choosing more aversive options also influenced attitude formation in certain types of evaluation, in which participants rated unhappiness on medical conditions as aversive events, and indicated preference change after choosing a medical condition they would rather avoid. As such, it would be possible to induce preference change after the blind choice task asking to choose more aversive options as in Experiment 2 if participants indicated their ratings of aversion for each facial stimulus in pre- and post-choice rating task. Thus, the compatibility between evaluation and choice decision might constrain the impact of choice on subsequent preference formation. Future studies should explore this in more detail. More broadly, the idea of compatibility between evaluation and choice decision could extend beyond choice-induced preference change. For instance, requiring participants to decide which face to be rounder could induce post-decisional change in facial roundness perception. It is possible that a variety of decisions alter relevant evaluation or perception after decision-making. This hypothesis awaits future testing.

### Stability of Updated Preference

The results from Experiment 4 demonstrated that preference change was still observed even after people explicitly knew their choice had been random. This raises the possibility that post-choice change in preference is not derived from consciously fitting preferences with choices in post-choice rating task, and that the updated preferences tend to be irrevocable once preference decision is made. These results are consistent with newer models suggesting that preference change occurs right at the moment of choice, thus immediately updating one’s preference [11], [33]. Although cognitive dissonance theory posits that options are revaluated after a choice is made with extended deliberation, choice-induced preference change might automatically emerge during the process of decision-making itself [40]. This assumption is supported by a neuroimaging study, which demonstrated that some parts of brain regions (e.g., inferior frontal gyrus, medial fronto-parietal regions and ventral striatum) related to preference change are rapidly engaged at the moment of choice decision [33]. Along with the finding, our data in Experiment 4 could be interpreted as evidence that an arbitrary choice of preferred face triggered revaluation immediately after making a choice and thus the updated preference through revaluation is stable even after knowing their choice was false. These findings indicate that psychological processes related to preference change can begin right at the moment of choice decision, not at the post-choice rating task.

Alternatively, a persistent impact of choice can be interpreted in terms of implicit choice memory [10], [11], [18]. As suggested by Lieberman and his colleagues [11], choice-induced preference change can rely on an automatic process that does not necessarily require explicit memory. In this assumption, evaluation in the post-choice rating can be affected by the stored choice memory without conscious retrieval of the choices. Extending this to our finding in Experiment 4, participants unwittingly incorporated arbitrary choices into revaluation in the post-choice preference rating, such that the impact of choice persists even though they explicitly knew that they did not have to reflect their choices in revaluation. The results from Experiment 4 emphasize that people are unaware of the causal origin of the updated preference in some cases.

### Conclusion

The results from the present four experiments demonstrated that randomly chosen faces were more preferred only after participants were required to choose “a preferred face,” but not “an unpreferred face,” and preference change was observed even after participants were informed that choices were actually random. Our results show important constrains under which random choice alters subsequent preference, suggesting different psychological processes involved in choice of preferred option and unpreferred one. Moreover, the impact of choice on preference formation may persist, once people make a choice even though it is actually random. This implies that people are tempted to make a biased evaluation with unaware of the causal origin of their own preference.
